# Integrated Immunomagnetic Bead-Based Microfluidic Chip for Exosomes Isolation

**DOI:** 10.3390/mi11050503

**Published:** 2020-05-15

**Authors:** Fuzhou Niu, Xifu Chen, Xuemei Niu, Yifan Cai, Qingkui Zhang, Tao Chen, Hao Yang

**Affiliations:** 1 School of Mechanical Engineering, Suzhou University of Science and Technology, Suzhou 215000, China; fzniu@usts.edu.cn (F.N.); xfchen@post.usts.edu.cn (X.C.); niuxm@mail.usts.edu.cn (X.N.); 2 Robotics and Microsystems Center, College of Mechanical and Electrical Engineering, Soochow University, Suzhou 215000, China; 20185229060@stu.suda.edu.cn (Y.C.); chent@suda.edu.cn (T.C.)

**Keywords:** exosomes isolation, immunomagnetic bead-based, microfluidic chip, micromixers

## Abstract

Exosomes are essential early biomarkers for health monitoring and cancer diagnosis. A prerequisite for further investigation of exosomes is the isolation, which is technically challenging due to the complexity of body fluids. This paper presents the development of an integrated microfluidic chip for exosomes isolation, which combines the traditional immunomagnetic bead-based protocol and the recently emerging microfluidic approach, resulting in benefits from both the high-purity of the former and the automated continuous superiority of the latter. The chip was designed based on an S-shaped micromixer with embedded baffle. The excellent mixing efficiency of this micromixer compared with Y-shaped and S-shaped micromixers was verified by simulation and experiments. The photolithography technique was employed to fabricate the integrated microfluidic chip, and the manufacturing process was elucidated. We finally established an experimental platform for exosomes isolation with the fabricated microfluidic chip built in. Exosomes isolation experiments were conducted using this platform. The distribution and morphology of the isolated exosomes were observed by transmission electron microscopy (TEM) and scanning electron microscopy (SEM). Quantitative size analyses based on transmission electron micrographs indicated that most of the obtained particles were between 30 and 150 nm. Western blot analyses of the isolated exosomes and the serum were conducted to verify the platform’s capability of isolating a certain subpopulation of exosomes corresponding to specified protein markers (CD63). The complete time for isolation of 150 μL serum samples was approximately 50 min, which was highly competitive with the reported existing protocols. Experimental results proved the capacity of the established integrated microfluidic chip for exosomes isolation with high purity, high integrity, and excellent efficiency. The platform can be further developed to make it possible for practical use in clinical applications as a universal exosomes isolation and characterization tool.

## 1. Introduction

Exosomes are nanosized (30–150 nm in diameter) extracellular vesicles (EVs) released from living cells which exist in almost all types of body fluids [1]. Exosomes are composed of substances derived from their originating cells, such as proteins, nucleic acids, lipids, and carbohydrates. They can merge with and then release their content into the surrounding cells via circulating fluids, thereby establishing intercellular communication and influencing various biological processes, including but not limited to, immune regulation, protein and/or lipid exchange, apoptosis, and antigen presentation [2]. Exosomes have recently been demonstrated to be involved in the development of cancer, wherein oncogenes exist. Therefore, exosomes can be used as potential early biomarkers for health monitoring and cancer diagnosis and treatment [3,4,5].

The development of a protocol capable of isolating exosomes with high purity, integrity, and efficiency is a prerequisite for the further investigation of exosomes [6,7]. However, exosomes isolation is technically challenging due to the complexity of body fluids, which contain diverse particles with similar size to exosomes. Numerous isolation protocols of different principles based on size differences among varying EVs or based on targeting specific surface markers, including ultracentrifugation, density gradient centrifugation, ultrafiltration, polymer precipitation, and immunoaffinity-based capture [8,9], have been reported. These exosomes isolation protocols have their own advantages, as presented in Table 1, and disadvantages, such as considerable time consumption (ranging from hours to days), high cost, relatively low exosome purity (mixed with other impurities), and relatively low exosome integrity (potentially causing structural damage).

Immunomagnetic bead-based exosomes isolation is one of the most common immunoaffinity-based capture methods [10]. Immunomagnetic beads are first produced by binding immunological ligands (such as antibodies) onto the magnetic beads’ surface, enabling the antigen-antibody specific reactions to capture specific corresponding exosomes. Typical exosome protein markers, such as CD9, CD63, and CD81, have been proven to distinguish exosomes and other EVs; thus, immunomagnetic beads coated with corresponding antibodies can specifically bind to exosomes by recognizing these protein markers. The target exosomes can be eluted and collected from the mixed bound substances, while the immunomagnetic beads are captured by the external magnetic field sourced by placed magnets/electromagnets. The steps of immunomagnetic bead-based exosomes isolation include (1) preparation of immunomagnetic beads (by binding specific antibodies to magnetic beads), (2) exosome capture using immunomagnetic beads, (3) impurity removal (while immunomagnetic beads with captured exosomes are held by the external magnetic field), and (4) exosome elution [11]. This immunomagnetic bead-based protocol allows the specific subpopulation of exosomes to be isolated and is particularly suited for small quantities of biological samples, resulting in benefits of high specificity, high purity, and zero damage to exosomes. However, the whole process is conducted manually, causing drawbacks, such as considerable time consumption, discontinuous multistep workflows, and influence due to personal experience differences. The mixing efficiency is evidently determined by manual vibration and can significantly affect the antigen–antibody specific reactions that bond the exosomes and immunomagnetic beads, which are the foundation for immunomagnetic bead-based exosomes isolation. 

The microfluidic chip is a recently emerging device employed in exosomes isolation [12,13]. Numerous studies combining physical (e.g., size and density) and/or biochemical (e.g., immunoaffinity) principles with the microfluidic technique have been explored to achieve rapid, efficient, low-cost, and automated exosomes isolation and overcome the drawbacks of traditional methods [14]. For instance, microfluidic chips can be designed in combination with sonic, electrophoretic, and electromagnetic forces to efficiently separate target exosomes [15,16,17,18,19]. 

Inspired by the high purity of the immunomagnetic bead-based protocol and the automated continuous superiority of microfluidic chips, this study aims to establish an efficient protocol for exosomes isolation. The proposed protocol combines the traditional immunomagnetic bead-based protocol and the recently emerging microfluidic approach. Three major attempts were made to achieve this goal, which designate this paper’s contributions, as listed below:

First, a microchannel-based mixer module was designed to improve the mixing efficiency of exosomes and immunomagnetic beads, which is the foundation in immunomagnetic bead-based exosomes isolation. On the basis of the flow characteristics and mixing mechanism of microfluids, we predesigned three types of micromixers with different microchannel structures (Y-shaped, S-shaped, and S-shaped with embedded baffle). The mixing performance of two-phase fluids inside each of the three micromixers was simulated and analyzed using Fluent software to evaluate the mixing efficiency; Meanwhile, the variance of the component concentrations in the cross-section of the channel was used as the evaluation index. Based on the simulation results, the S-shaped micromixer with embedded baffle was consequently selected to further fabricate a microfluidic chip for exosomes isolation. Additionally, experiments were conducted to verify the mixing efficiency of the proposed micromixer. The experimental results matched well with the simulation results.

Second, a microfluidic chip was designed and fabricated for high-efficiency and automated continuous exosomes isolation. 

Corresponding to three specific procedures of traditional immunomagnetic bead-based exosomes isolation (namely, binding of immunomagnetic beads and exosomes, impurities cleaning, and elution and collection of exosomes), we designed a microfluidic chip composed of three inlets, one outlet, three mixers, and three chambers based on the S-shaped micromixer with embedded baffle. The capture capability of a magnet with a diameter of 4 mm was experimentally evaluated and verified; And, three such magnets were placed on the three chambers to generate the required magnetic field for capturing immunomagnetic beads and/or the bound immunomagnetic beads and exosomes. Besides, the manufacturing process of the microfluidic chip was elucidated.

Third, we developed an exosomes isolation system with the fabricated microfluidic chip built in. Exosomes isolation experiments were conducted using this system. Detailed procedures of exosomes isolation were described. The experimental results demonstrated the system’s capacity of continuous automated exosomes isolation with high purity, high integrity, and excellent efficiency. The system can be further used in concentration calibration and morphological representation of exosomes.

## 2. Micromixer Design

In the manipulation of immunomagnetic bead-based microfluidic exosomes isolation, mixing sufficiency is crucial for binding immunomagnetic beads and exosomes [20]. The microchannel-based mixer is a recently emerging device for rapid and complete mixing; it can offer remarkable benefits over conventional mixing methods, such as reagent reduction, low cost, enhanced sensitivity, and high efficiency. As the fluid flow in micro-sized channels is inherently laminar, the mixing of fluids remains a challenge. The microchannel-based mixer can be categorized into passive and active to enhance the mixing performance. However, the active micromixer is complex in fabricating procedures and requires external enforcing energy. Therefore, a passive micromixer is utilized in our design. The typical passive methods employed to enhance mixing efficiency predominantly rely on chaotic convection effects by handling flows within the microchannels or by improving diffusion via (1) increasing the contact area and/or time among mixing species [20], or via (2) influencing the flow streamlines to enhance dispersion-caused mass transfer [21,22,23]. Taking the microscale fluid characteristics and mixing performance into account, three specified microchannel structures were studied and compared by numerical simulation using Ansys Fluent software to select the most suitable one for the fabrication of a microfluidic chip for exosomes isolation.

**Figure 1:**
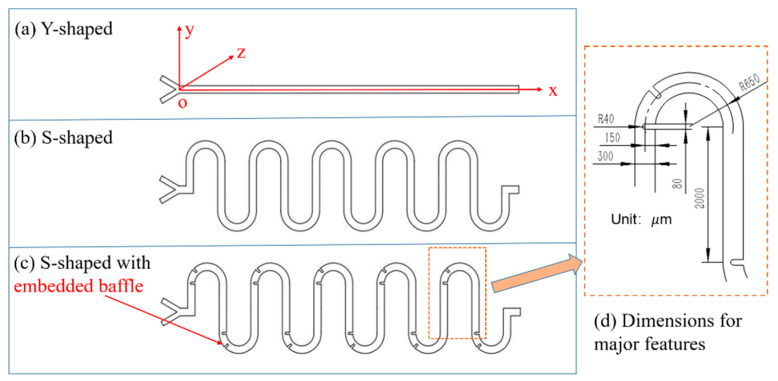
Three types of microchannel-based mixers. (**a**) Y-shaped: straight microchannels with no meander structure. (**b**) S-shaped: meander microchannels for increasing contact area/time of diffusion. (**c**) S-shaped with embedded baffle: meander microchannels with embedded baffle obstacles for inducing chaotic convection effects by altering the geometric surface of the microchannels. (**d**) Dimensional drawing for major structural features.

The schematic of the three microchannel structures for simulation experiments, namely, (a) Y-shaped, (b) S-shaped, and (c) S-shaped with embedded baffle, is shown in Figure 1a–c, where the origin O and the 2D x–y coordinates are plotted, and the z-axis is in the direction of the microchannel depth, which is fixed at 50 μm. The S-shaped channel and the embedded baffle were designed to achieve better mixing efficiency while reducing operation time, benefiting from longer mass transferring channel length and stronger forced and intersected flow streamlines. The width of all microchannel types was 300 μm, the dimensions for other major structure features are illustrated in Figure 1d. The total channel length is $7\times 10^{3}$ μm for the Y-shaped mixer, and $\sim 3.9\times 10^{4}$ μm for the other two mixers. Each channel had two inlets and one outlet. Two fluids, deionized (DI) water and ethanol at 20 °C, were assigned into each inlet in the numerical simulation, whose parameters are given in Table 2. The fluids were driven by applying pressure between inlets and outlet using syringe pumps. The pressure on inlets varied with the velocity of introduced fluids, whereas the pressure on the outlet remained static zero. Boundary conditions of the inlet, outlet, and microchannel’s inner wall were set as laminar inflow, laminar outflow, and no-slip boundary, respectively. We used the Reynolds number (*Re*) as the independent variable throughout the simulation to observe the mixing performance. The *Re* of fluid flow at microscale is generally less than 100. Thus, *Re* of no more than 100 was adopted in the simulation (*Re* = 1, 10, 50, 100). Corresponding to a specified *Re*, the inlet velocity of each fluid can be calculated based on the following mathematical relationship:

$$Re=\frac{\rho \nu d}{\mu }$$

where ρ is the density of the fluid, ν is the relative velocity of the fluid to the microchannel, and μ is the dynamic viscosity of the fluid.

**Figure 2:**
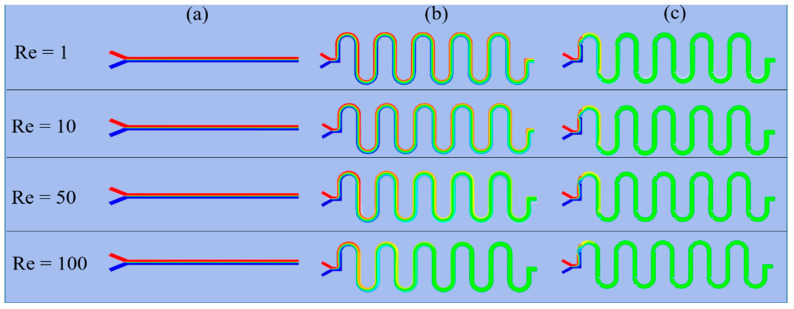
Numerical concentration distribution results of two-phase mixed fluid flow within each micromixer in the x–y plane for different Re numbers (*Re* = 1, 10, 50, 100): (**a**) Y-shaped, (**b**) S-shaped, and (**c**) S-shaped with embedded baffle. Red, corresponding to *Vf* = 1, denotes that the microchannel is full of the red-colored solution at the specified point. Blue, corresponding to *Vf* = 0, denotes that the microchannel is full of the blue-colored solution but no red-colored solution. Green represents complete mixing of the two-phase fluid, with *Vf* = 0.5.

The simulated mixing performance of each micromixer with varying *Re* is shown in Figure 2. The mixing performance was evaluated using a quantitatively standard mixing index [24,25], which presents the standard deviation of the mixture concentrations of the whole sampling points in the middle cross-section of the channel parallel to the x–y plane. The mixing index *M* is expressed as follows:

$$M=1-\frac{\sigma }{\sigma_{\max}}$$

$$\sigma =\sqrt{\frac{1}{N}\sum_{i=1}^{N}(C_{i}-\bar{C}{)}^{2}}$$

where *M* (0 ≤ *M* ≤ 1) is the mixing index; *σ* is the standard deviation of concentration; *σ*_max_ is the maximum value of σ; $C_{i}$ and $\bar{C}$ are the concentration values at *i*th sampling point and the average concentration of the total sampling points, respectively; and *N* represents the number of sampling points on the observed cross-section.

Especially, *M* = 0 denotes no mixing (in the case of *σ* = *σ*_max_), whereas *M* = 1 denotes complete mixing (in the case of *σ* = 0). Increasing mixing index *M* corresponds to well-distributed mixed fluids. The mixing index represents the mixing efficiency, that is, the larger *M*, the better distributed is the mixing efficiency.

The mixing index was studied by analyzing the simulated concentration distribution. The concentration profile was obtained by using commercial Ansys Fluent software. The governing equations for solving the simulation involved the Navier–Stokes, continuity, and convection-diffusion equations. Steady-state, Newtonian, and incompressible fluidic conditions were assumed. The ICEM preprocessor was applied to generate meshes; the smallest cell size was 12.5 microns. Software built-in hexahedral meshing was utilized to generate computational cells. The SIMPLEC algorithm was used to deal with the coupling problem of pressure and velocity. After the computational task was completed, a CFD-Post module was used for post-processing of the solution results.

Figure 2 shows the detailed numerical concentration distribution results of two-phase mixed fluid flow at the middle cross-section within each micromixer in the x–y plane for different *Re* (*Re* = 1, 10, 50, 100). The two-phase fluids are initially marked with blue and red to enhance visualization. For convenience, we defined the volume fraction of the originally red-colored solution as *V_f_*. Therefore, the mixing efficiency of the two-phase fluids was evaluated by the plotted color. The two-phase fluid was completely mixed when the color distribution turned green (in case of *V_f_* = 0.5, corresponding to *M* = 1.0). From Figure 2, we can intuitively evaluate that the S-shaped micromixer with embedded baffle had the best mixing efficiency, followed by the S-shaped micromixer, whereas the Y-shaped micromixer exhibited poor mixing efficiency. 

**Figure 3:**
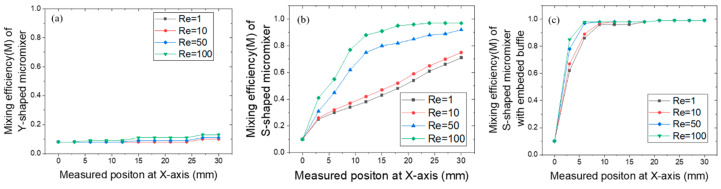
Quantitative mixing efficiency of the three micromixers along the x-axis at different Res: (**a**) Y-shaped, (**b**) S-shaped, and (**c**) S-shaped with embedded baffle.

The detailed quantitative post-processing results of mixing efficiency, measured using the mixing index, are shown in Figure 3. The concentration profile within the Y-shaped micromixer was almost consistent for varying *Re* numbers at any observed cross-section perpendicular to the x-axis along the length (from the x-position of the inlet to that of the outlet), resulting in *M* = 10% mixing efficiency at the outlet. For the S-shaped micromixer, the mixing efficiency significantly improved as *Re* increased. If *Re* was greater than 50, then the fluids could achieve complete mixing ahead of the outlet. However, at a low *Re*, the fluids could not be completely mixed even at the outlet. Apparently, the S-shaped micromixer with embedded baffle showed remarkable mixing efficiency. Despite the varying *Re*, complete mixing of the fluids can be accomplished within a fairly short microchannel length.

As a result, the S-shaped micromixer with embedded baffle was selected to fabricate an integrated microfluidic chip for exosomes isolation.

## 3. Microfluidic Chip Fabrication

**Figure 4:**
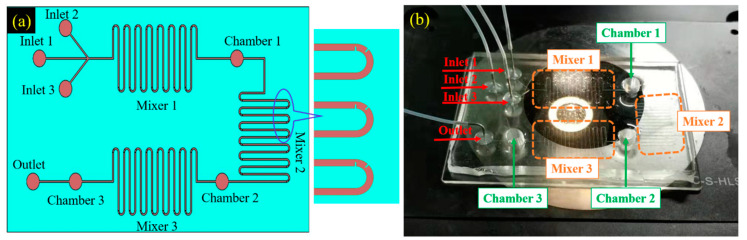
Designed microfluidic chip for exosomes isolation, (**a**) design schematic, and (**b**) fabricated chip.

A microfluidic chip was designed based on the S-shaped micromixer with embedded baffle to ensure rapid and efficient exosomes isolation. The schematic of the chip is shown in Figure 4a, and the fabricated chip is shown in Figure 4b. The chip included three inlets, one outlet, three mixers, and three chambers. The channel of each mixer was 30 mm long at the x-axis, 300 microns wide, and 50 microns high. Three magnets with a diameter of 4 mm were placed on each of the three chambers to generate an external magnetic force for capturing immunomagnetic beads and/or bound immunomagnetic beads and exosomes. The photolithography technique was employed to fabricate the chip in a clean room, whose detailed processing is presented as follows:

### 3.1. Producing the Microchannel Mold

The designed microchannels were patterned on a single-side silicon wafer, which was cleaned beforehand by an ultrasonic water bath with ethanol, acetone, and isopropanol solution successively for 20 min and dehydrated with nitrogen flow stream. The photoresist was coated on the prepared silicon wafer by a KW-4A spin coater at 1600 rpm/s to achieve the required height of microchannels. The wafer coated with SU-8 (SU-8 2050, Microchem Corp., Westborough, MA, USA) film was preheated using a heating plate for 1min at 65 °C and 7 min at 95 °C. Preheating was undertaken to enhance the wear resistance between the photoresist and the substrate to withstand the friction between the mask and the film during the next exposure processing. The coated wafer was exposed to 365 nm UV light for 16s. The heating plate was used for post-heat treatment for 1 min at 65 °C and 7 min at 95 °C. The wafer was placed into a Petri dish filled with developing solution (RZX-3038, Suzhou Ruihong Electronic Chemical Co., Ltd., Suzhou, China) and shaken for 2 min. Eventually, a microchannel mold with 50 μm height was produced.

### 3.2. Fabricating the Microchannel Structure

Once the silicon mold was produced, the completely mixed PDMS (Sylgard 184, Dow Corning, Midland, MI, USA) and curing agent solution with a mass ratio of 10:1 was poured onto the mold. Three thin cylindrical rods with a diameter of 5 mm and a length of 6 mm were implanted into the PDMS to fabricate the three chambers, whose terminals were parallel to but did not touch the surface of the silicon wafer. All subjects were cured at 85 °C for 4 h in a vacuum drying oven. After curing, the PDMS layer containing the micro-channel structure was slowly torn off along the interface between PDMS and the mold.

### 3.3. Bonding Microchannel to Glass Substrate

The three inlets and one outlet were drilled in advance using a Uni-core punch with an inner diameter = 1.5 mm (Model:WH-CF-13, Wenhao Co., Ltd., Suzhou, China). The obtained PDMS layer was then bound to a glass slide. The slide was precleaned by an ultrasonic water bath with ethanol, acetone, and isopropanol solution successively for 10 min, rinsed well with DI water, and dehydrated using nitrogen flow stream. The bonding processing included oxygen plasma treatment (PDC-32G-2 plasma cleaner, Harrick Plasma Company, Ithaca, NY, USA) for 3 min to form the integrated microfluidic chip. Stainless steel injection needles were connected to PTFE tubes (0.5 mm inner diameter) and inserted into the predrilled inlets/outlet holes of the chip. Sealing was conducted around the inlets/outlet holes to strengthen the tightness between the stainless steel needles and the chip.

## 4. Experimental

**Figure 5:**
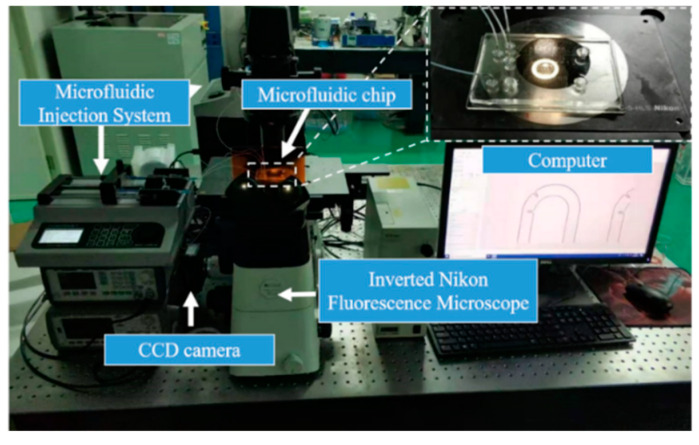
Integrated experimental platform for exosomes isolation.

The integrated experimental platform for exosomes isolation, which included a microfluidic injection system, an inverted Nikon fluorescence microscope, a CCD camera, a microfluidic chip, and a computer, is depicted in Figure 5. The microfluidic injection system was used to control the speed and volume of injection. The CCD camera and Nikon fluorescence inverted microscope were used to (1) observe the flow of liquid in the microfluidic chip and the process of magnetic bead capture and (2) record the experimental results in real-time.

### 4.1. Magnetic Capturing Capability Verification

**Figure 6:**
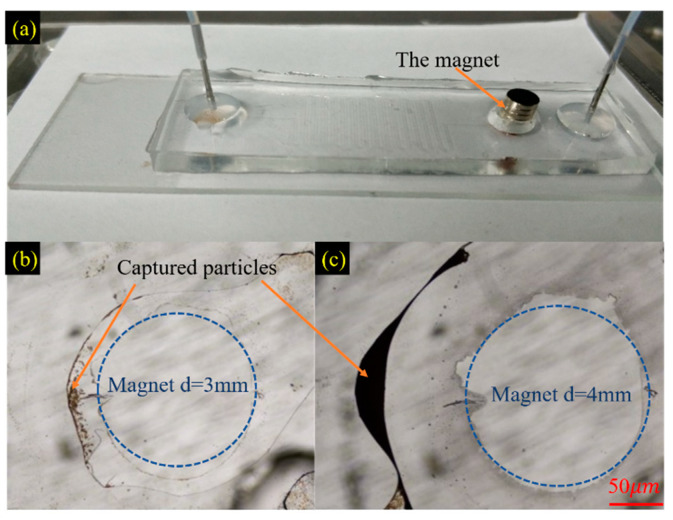
(**a**) Microfluidic chip of a simple S-shaped micromixer with embedded baffle to verify the magnetic capture capability and the capturing performance of magnets with diameters of (**b**) 3 mm and (**c**) 4 mm within the chamber.

According to the steps of immunomagnetic bead-based exosomes isolation mentioned in Introduction, particles (such as immunomagnetic beads and/or bound immunomagnetic beads and exosomes) should be captured and released by the ON/OFF situation of external magnetic fields. To demonstrate the capturing capacity of the placed magnets on the chambers, experiments were performed using silica magnetic microspheres (Affimag SLC, BaseLine Chromtech, Tianjin, China) with 2.8 μm diameter (100 mg/10 mL) as samples. In these experiments, a simple S-shaped micromixer with embedded baffle was utilized, containing one inlet, one outlet, and one chamber, as illustrated in Figure 6a; And, the channel of the micromixer was 30 mm length, 300 μm width, and 50 μm height. The channel parameters of the experimental micromixer were matched with the integrated microfluidic chip for exosomes isolation. 

**Figure 7:**
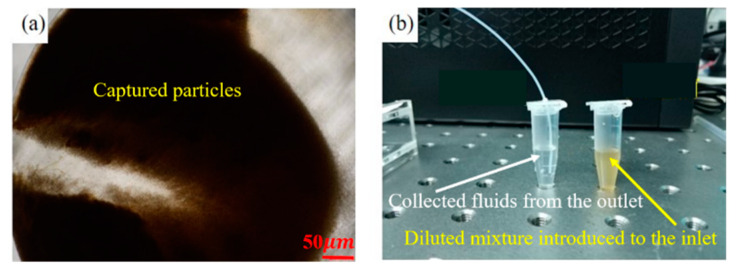
After 50 min of manipulation, (**a**) the capture performance was considerably enhanced and (**b**) the collected fluids from the outlet were clear compared with the diluted mixture introduced into the inlet.

The magnetic silica microsphere suspension was diluted 10 times with PBS solution in advance. The diluted mixture was dispersed by an ultrasonic cleaner to reduce the agglomeration of microparticles. The mixture was introduced into the inlet with a flow rate of 20 μL/min (with a corresponding Reynolds number about 66.7) using a syringe pump. Magnets with diameters of 3 and 4 mm were placed onto the chamber to evaluate the capture capacity. The capturing results after 5 min are shown in Figure 6b,c. The magnet with a diameter of 4 mm showed better-capturing performance (Refer to the Appendix A for the comparison of the generated magnet field distribution using the two magnets.). Furthermore, taking into account that the chamber has a diameter of 5 mm, the magnet with a diameter of 4 mm was chosen to generate the required magnetic field for subsequent tasks. On increasing the time of the capturing process (~50 min), the capture performance of the 4 mm magnet was highly improved (as shown in Figure 7a). This process resulted in clear PBS solution collected in the outlet, compared to the introduced turbid mixture in the inlet (as shown in Figure 7b).

The captured magnetic silica microparticles could be released from the chamber by continuously introducing PBS solution. With an increase in elution time, the particles could be well eluted. However, some particles remained in the chamber due to the nonspecific adsorption between the particles and the PDMS surface. Fetal bovine protein serum (BSA) is usually used to pretreat the microfluidic chips to reduce nonspecific adsorption.

### 4.2. Mixing Performance Demonstration

**Figure 8:**
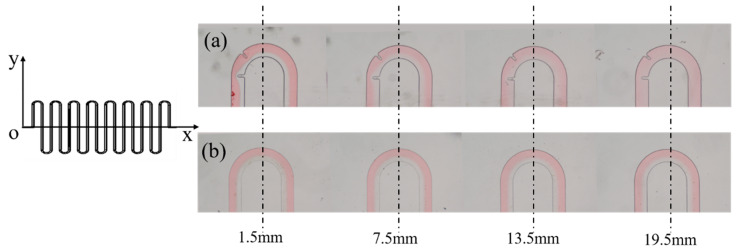
Experimental results of mixing performance of (**a**) S-shaped micromixer with embedded baffle and (**b**) S-shaped micromixer. The evenness of the red colored distribution indicates the mixing efficiency.

The simulation results confirmed that the S-shaped micromixer with embedded baffle exhibited the best mixing performance among the three proposed micromixers, and the S-shaped micromixer had the second-best mixing performance. Here, experiments were conducted using these two fabricated micromixers. Ethanol and DI water were used as experimental samples. Ethanol was dyed with red ink to improve visualization. Each of the two inlets was injected with ethanol and DI water using two syringe pumps. A CCD camera and an inverted Nikon fluorescent microscope were utilized to establish the imaging process for observing, as shown in Figure 5. DI water and ethanol were placed in a vacuum environment for 30 min to remove air bubbles and then introduced into each inlet of the micromixer simultaneously with the flow rate of the two syringe pumps set at 20 μL/min (with a corresponding Reynolds number about 66.7). A long processing time (approximately 20 min) was needed to ensure the stability of the flow and mixing for image data collection. Figure 8 shows the experimental results. The results were in excellent agreement with the simulation results, thereby demonstrating the mixing efficiency of the S-shaped micromixer with embedded baffle.

### 4.3. Exosomes Isolation

**Figure 9:**
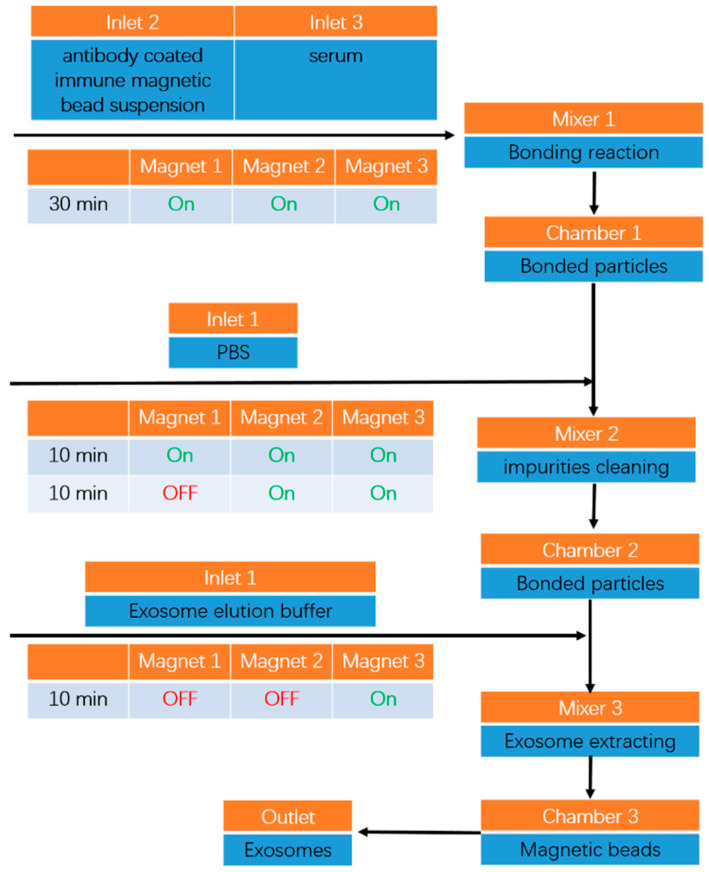
Processing flow chart of exosomes isolation using the designed microfluidic chip. The green “on” represents the magnet corresponding to *i*th chamber, and the red “off” is moved off. The times indicate that this step of the operation should last for that long. The blue blocks indicate the relevant substances enriched or the procedures that occurred within the corresponding substructures (marked with orange above each blue block) of the microfluidic chip.

The process diagram of exosomes isolation using the designed microfluidic chip is shown in Figure 9. 

The first step was the binding of immunomagnetic beads and exosomes. Inlets 2 and 3 were injected with antibody-coated immune magnetic bead suspension and serum using syringes at a flow rate of 5 μL/min, lasting for 30 min. The two solutions were completely mixed in Mixer 1 for bonding reaction (specific antigen-antibody reaction). When the mixed liquid flowed through the chambers, the enriched particles (the bound magnetic beads and exosomes) were captured by the placed magnets. The majority of the bound particles were restricted within Chamber 1, whereas the rest were captured by magnets on Chambers 2 and 3. Additional fluids were introduced into Inlets 2 and 3 until the desired amounts of immune magnetic bead suspension and serum solution were injected. 

The second step was impurity cleaning. PBS was injected into Inlet 1 at a flow rate of 10 μL/min for 10 min, and then the magnet placed on Chamber 1 was removed. The injection of PBS solution into Inlet 1 was continued for an additional 10 min to flush impurities that remained in the channel. During the cleaning process, bound magnetic beads with exosomes and PBS solution were completely mixed within Mixer 2 and then captured by the magnet placed on Chamber 2.

The third step was exosomes isolation and collection. An exosome elution buffer containing the chelating agent EDTA was injected into Inlet 1 at a flow rate of 5 μL/min for 10 min. The magnet placed on Chamber 2 was removed. Bound magnetic beads with exosomes and elution buffer were completely mixed within Mixer 3. The exosomes were eluted and flushed out of the outlet for collection, and the magnetic beads were captured by the magnet placed on Chamber 3.

The complete time for isolation of 150 μL serum samples was approximately 50 min, which was highly competitive with the reported existing protocols.

In this study, the serum of healthy mice was selected as the sample for exosomes isolation. The serum was extracted through a refrigerated high-speed centrifuge using a centrifuge (Thermo Fisher Scientific, Waltham, MA, USA) from the original blood provided by the medical department of Soochow University. (Refer to Appendix B for the specific operation process of the refrigerated high-speed centrifuge.) The immunomagnetic beads used in the experiment were made by a combination of biotin-labeled exosome capture antibodies (with CD63 antigen) and streptomycin affinity magnetic beads, both of which were commercially purchased (Realgen-bio Co., Ltd., Shanghai, China, refer to Appendix C for the detailed preparation procedures for immunomagnetic beads). 

### 4.4. Characterization of Isolated Exosomes

Transmission electron microscopy (TEM) is a popular technique for the characterization of exosomes; it has excellent resolution and is capable of imaging objects of less than 1 nm [26,27]. Heavy metal substances, such as phosphotungstic acid and uranium acetate, can be used for contrast with lipid membranes to facilitate the observation of vesicle structures. 

In this study, the isolated exosomes were mixed with a 4% paraformaldehyde solution at a ratio of 1:1; 10 μL of the mixture was absorbed using a pipette and dropped on a sample nickel grid. The sample nickel grid was placed at room temperature for 15 min for complete absorption. Paraformaldehyde was used to fix the morphology of the exosomes. Then, 5 μL of 1% phosphotungstic acid was dropped onto the nickel grid for negative staining of the exosomes for 5 min. Finally, the extra liquid was absorbed by filter paper to complete exosome sample preparation for TEM observation.

**Figure 10:**
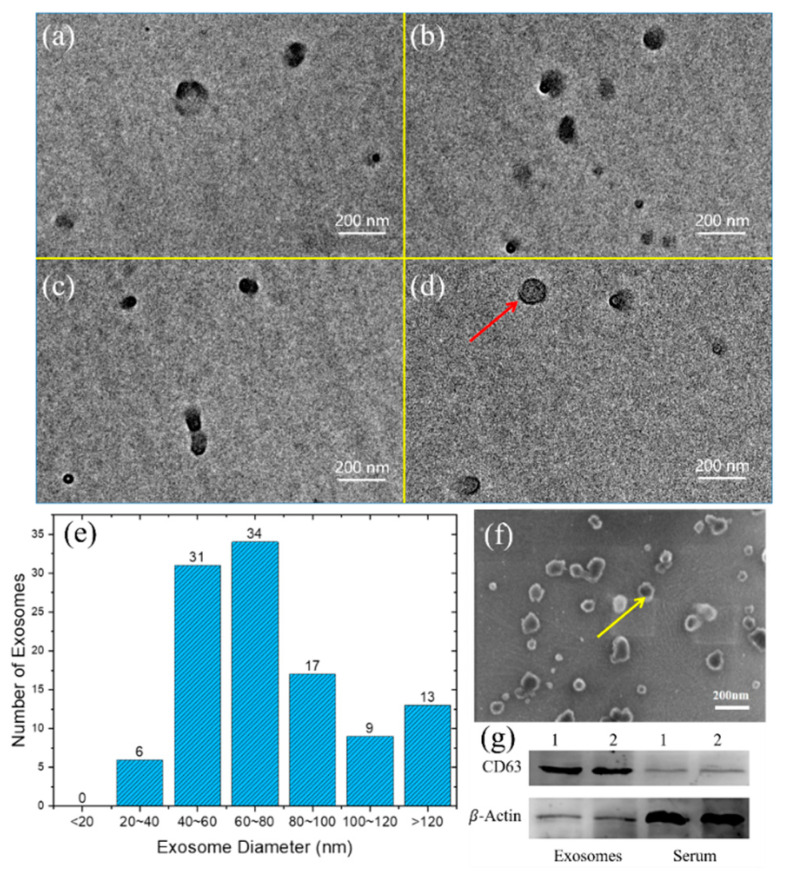
Identification and characterization of isolated exosomes using (**a**–**d**) TEM. Red arrow: the isolated exosomes contain typical membrane structure after negative dyeing treatment. (**e**) Quantitative size analyses: more than 95% of the obtained particles are between 30 and 150 nm. (**f**) SEM. Yellow arrow: round bowl-shaped morphology of the isolated exosomes can be observed. (**g**) Western blot. Western blot of CD63 and β-actin protein expression in isolated exosomes and serum lysates from two samples.

The isolated exosomes were visualized by TEM and scanning electron microscopy (SEM) to confirm that the isolated particles were “exosomes” but not other EVs. Figure 10a–d illustrate the distribution and morphology images of the isolated exosomes using TEM captured at four different viewing fields. The images show that the isolated exosomes exhibited high integrity (no other proteins, large vesicles, or other impurities). The obtained particles contained a typical membrane structure after negative dyeing treatment, as depicted in Figure 10d. Quantitative size analyses of the isolated exosomes using ImageJ software at 20 different TEM viewing fields are shown in Figure 10e. Over 95% of the obtained particles were between 30 and 150 nm, which was consistent with the known size characterization of exosomes [1]. SEM images in Figure 10f were used to observe the morphology of isolated exosomes. A clear round bowl-shaped morphology of the isolated exosomes was observed ranging from 30 nm to 150 nm in diameter, which was consistent with the measured results of TEM. However, the exosome measurement results obtained by SEM were inadequate to characterize the exosome membrane structure. The universal identification of exosomes is commonly based on their size and membrane composition [28]. Thus, the obtained substances were confirmed to be exosomes. 

Western blot analyses of the isolated exosomes and serum were conducted after lysis with RIPA buffer (Realgen-bio Co., Ltd., Shanghai, China). Figure 10g shows that tetraspanin CD63 was highly harvested in the isolated exosomes but barely detectable in the whole-cell lysate of serum. The ability to isolate a certain subpopulation of exosomes corresponding to specified protein markers (CD63) was verified.

All these experimental results demonstrated the capacity of the established integrated microfluidic chip for exosomes isolation with high purity, high integrity, and excellent efficiency. 

## 5. Conclusions and Discussion

In this study, we developed an integrated chip for exosomes isolation, which combined the traditional immunomagnetic bead-based protocol and the recently emerging microfluidic approach. The chip benefitted from the high purity of the immunomagnetic bead-based protocol and the automated continuous superiority of microfluidic chips. The chip was designed based on an S-shaped micromixer with embedded baffle, which exhibited excellent mixing efficiency, verified by simulation and experiments. Simulated concentration distribution results of two-phase mixed fluid flow within three different micromixers were investigated and compared. The S-shaped micromixer with embedded baffle was selected to fabricate the proposed chip for exosomes isolation. The mixing efficiency of the S-shaped micromixer with embedded baffle was also demonstrated by observing the mixing performance of introduced DI water and ethanol within the mixer. The results were in excellent agreement with the simulation results.

The integrated chip included three inlets, one outlet, three mixers, and three chambers. The channel of each mixer was 30 mm long at the x-axis, 300 microns wide, and 50 microns high. Three magnets with a diameter of 4 mm were placed on each of the three chambers to generate an external magnetic force for capturing immunomagnetic beads and/or bound immunomagnetic beads and exosomes. The placed magnets’ capabilities of capturing and releasing magnetic particles were experimentally verified. A photolithography technique was employed to fabricate the integrated microfluidic chip, and the manufacturing process was elucidated.

We finally established an experimental platform for exosomes isolation with the fabricated microfluidic chip built in. The platform contained a microfluidic injection system, an inverted Nikon fluorescence microscope, a CCD camera, the microfluidic chip, and a computer. Exosomes isolation experiments were conducted. The experimental results of the distribution and morphology of isolated exosomes were observed by TEM and SEM. Quantitative size analyses based on transmission electron micrographs indicated that over 95% of the obtained particles were between 30 and 150 nm in diameter. Western blot analyses of the isolated exosomes and serum were conducted to verify the platform’s capacity to isolate exosomes corresponding to specific protein markers (CD63). The complete time for isolation of 150 μL serum samples was approximately 50 min. These results demonstrated the capacity of the developed integrated platform for exosomes isolation with high purity, high integrity, and excellent efficiency. The platform can be potentially enhanced for practical biomedical use as a universal exosomes isolation and characterization instrument. The proposed approach of exosomes isolation could not only contribute to a better understanding of cell pathology and physiology within the coming several years, but also assist in the diagnosis and prognosis of many diseases like neurodegenerative disorders, cancer, and AIDS in future.

Some points need to be improved in future work. First, in this version of the designed microfluidic chip, the fabrication of chambers is relatively complicated, and magnets are manually operated to be placed on or removed from the chambers. The magnet should be updated to an electromagnet, which can enable automated capture and release of magnetic particles. Second, the minimum sample of serum for exosomes isolation is restricted to 50 μL due to the size of the fabricated microfluidic chip. A more refined microchannel structure needs to be designed and fabricated for precise exosomes isolation using a lower sample dosage.

## Figures and Tables

**Table 1:** Comparison of traditional exosomes isolation methods.

Isolation Protocol	Principle of Separation	Advantages	Disadvantages
Ultracentrifugation	Density, size	Large batch separation	Considerable time consumption High cost Potential structural damage
Polymer precipitation	Polymer precipitation	Good specificity, Large batch separation, No need for large instruments	Mixed with other impurities Long processing time
Ultrafiltration	Size	No reagent/label contamination, No damage to exosomes	Mixed with other similar-sized impurities
Immunoisolation	Antigen-antibody reaction	High specificity and purity	Multiple steps Require trained personnel Inefficient

**Table 2:** Parameters of fluids in the simulations.

Fluids	Density (kg/m^3^)	Viscosity (Pa·s)	Diffusivity (m^2^/s)
DI water	9.998 × 10^2^	0.9 × 10^−3^	1.2 × 10^−9^
Ethanol	7.89 × 10^2^	1.2 × 10^−3^	1.2 × 10^−9^

## Appendix A. Comparison of the Distribution of the Two Magnets

The maximum magnitude of the generated magnetic field flux and gradient using a magnet (with a diameter of 3- or 4-mm and a same thickness of 1 mm, NdFeB35 material) is nearly the same (about 0.3 T and 800 T/m). The results are generated in a square area in a parallel plane to the bottom of the magnet with a distance of 0.2 mm, while the effective working region of the 4 mm-diameter magnet is much larger than the 3 mm-diameter magnet, enabling better isolation performance [29,30].

## Appendix B. Preparation of the Serum Sample

(1) The original blood was placed into a non-anticoagulant tube and stored for 4 h in refrigerated mode, then the precipitated supernatant liquor was the serum sample. The supernatant was collected.

(2) The supernatant in (1) was transferred to a new centrifuge tube and centrifuged for 20 min at 1200× *g* in a refrigerated high-speed centrifuge (to separate cells). The supernatant was collected.

(3) The supernatant in (2) was transferred to a new centrifuge tube and centrifuged for 20 min at 10,000× *g* in a refrigerated high-speed centrifuge (to separate large EVs). The supernatant was collected.

(4) The supernatant in (3) was transferred to a new centrifuge tube. The supernatant was collected.

The obtained supernatant in (4) was the serum sample.

## Appendix C. Preparation Procedures of Immunomagnetic Beads

(1) The magnetic beads were completely mixed using a vortex mixer; 50 µL of the mixed magnetic bead suspension was absorbed and moved to a centrifuge tube. The centrifuge tube was placed on a magnetic grate for 2 min of magnetic separation. A pipette was used to remove the supernatant.

(2) Exosome immobilization buffer (500 μL) was added to the centrifuge tube (the same as in (1)), and the buffer was mixed using the vortex mixer. The centrifuge tube was placed on a magnetic grate for 2 min magnetic separation. A pipette was used to remove the supernatant.

(3) Exosome immunofixation buffer (500 μL) and biotin-labeled exosome capture antibodies (10 μL) were added to the centrifuge tube. The mixture was completely mixed using the vortex mixer and then held at 24 °C for 20 min.

(4) Step (2) was repeated twice to complete the preparation of immunomagnetic beads.
